# Bis(tetra­butyl­ammonium) [2-(eth­oxy­carbony)phenyl­imido]-μ_6_-oxido-dodeca-μ_2_-oxido-penta­oxidohexa­molybdenum diethyl ether hemisolvate

**DOI:** 10.1107/S1600536812026323

**Published:** 2012-06-16

**Authors:** Manxiang Wang, Shikai Tao, Jing Guo, Jian Hao, Qiang Li

**Affiliations:** a Department of Chemistry, College of Science of Beijing Forestry University, Beijing 100083, People’s Republic of China; b Beijing Petroleum Machinery Factory, Beijing 100083, People’s Republic of China; c Liangxi Science Experimental Class of Beijing Forestry University, Beijing 100083, People’s Republic of China; d Department of Chemistry, College of Science of Beijing University of Chemical Technology, Beijing 100029, People’s Republic of China

## Abstract

In the title complex, [(C_4_H_9_)_4_N]_2_[Mo_6_O_18_(C_9_H_9_NO_2_)]·0.5C_4_H_10_O, the aryl­imido ligand is linked to an Mo atom of the Lindqvist-type polyoxidometalate anion by an Mo N bond of 1.726 (4) Å. The Mo N—C angles are 160.7 (5) and 167.6(5)° because of disorder affecting the aryl group, and is typical for the imido monodentate behaviour described in analogous hybrids. Light components of the structure are extensively disordered. The aryl ester group is disordered over two positions with occupancies refined to 0.559 (3) and 0.441 (3). Both independent tetra­butyl­ammonium cations have butyl chains partially split over two sites, with occupancies as in the aryl group of the anion. Finally, the ether solvent mol­ecule is disordered around an inversion centre. In the crystal, cations and anions inter­act *via* C—H⋯O contacts, involving O atoms of the polyoxidometalate anion and the ester group of the aryl­imido ligand as acceptor groups.

## Related literature

For general background to polyoxidometalates and the synthesis of their organoimido derivatives, see: Hill (1998 ▶); Li *et al.* (2011 ▶); Du *et al.* (1992 ▶); Mohs *et al.* (1995 ▶); Clegg *et al.* (1995 ▶); Wu *et al.* (2004 ▶). For structural features characteristic of these complexes, see: Karlin & Wigley (2007 ▶); Li *et al.* (2008 ▶). 
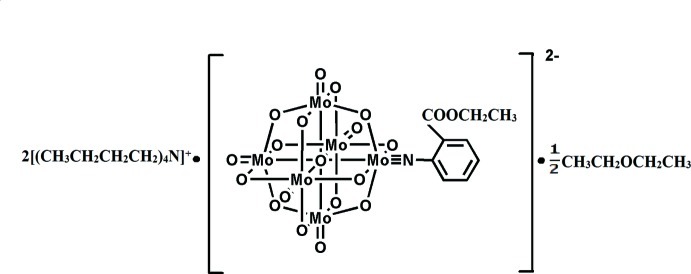


## Experimental

### 

#### Crystal data

(C_16_H_36_N)_2_[Mo_6_O_18_(C_9_H_9_NO_2_)]·0.5C_4_H_10_O
*M* *_r_* = 1548.79
Monoclinic, 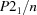

*a* = 16.3974 (16) Å
*b* = 17.0854 (8) Å
*c* = 21.687 (3) Å
β = 106.709 (16)°
*V* = 5819.1 (10) Å^3^
*Z* = 4
Mo *K*α radiation
μ = 1.33 mm^−1^
*T* = 101 K
0.40 × 0.30 × 0.25 mm

#### Data collection

Agilent Xcalibur Eos Gemini diffractometer
Absorption correction: multi-scan (*CrysAlis PRO*; Agilent, 2011 ▶) *T*_min_ = 0.619, *T*_max_ = 0.733
31709 measured reflections
11422 independent reflections
8774 reflections with *I* > 2σ(*I*)
*R*_int_ = 0.038

#### Refinement

*R*[*F* ^2^ > 2σ(*F* ^2^)] = 0.039
*wR*(*F* ^2^) = 0.088
*S* = 1.05
11422 reflections
854 parameters
500 restraints
H-atom parameters constrained
Δρ_max_ = 1.46 e Å^−3^
Δρ_min_ = −0.81 e Å^−3^

### 

Data collection: *CrysAlis PRO* (Agilent, 2011 ▶); cell refinement: *CrysAlis PRO*; data reduction: *CrysAlis PRO*; program(s) used to solve structure: *SHELXS97* (Sheldrick, 2008 ▶); program(s) used to refine structure: *SHELXL97* (Sheldrick, 2008 ▶); molecular graphics: *DIAMOND* (Brandenburg, 2010 ▶); software used to prepare material for publication: *publCIF* (Westrip, 2010 ▶).

## Supplementary Material

Crystal structure: contains datablock(s) I, global. DOI: 10.1107/S1600536812026323/bh2428sup1.cif

Structure factors: contains datablock(s) I. DOI: 10.1107/S1600536812026323/bh2428Isup2.hkl

Additional supplementary materials:  crystallographic information; 3D view; checkCIF report

## Figures and Tables

**Table 1:** Hydrogen-bond geometry (Å, °)

*D*—H⋯*A*	*D*—H	H⋯*A*	*D*⋯*A*	*D*—H⋯*A*
C3*A*—H3*A*⋯O19^i^	0.93	2.38	3.287 (9)	164
C5—H5⋯O7^ii^	0.93	2.58	3.449 (14)	155
C21—H21*B*⋯O15^i^	0.97	2.43	3.354 (5)	159
C25—H25*A*⋯O6^iii^	0.97	2.58	3.544 (5)	173
C41—H41*B*⋯O14	0.97	2.56	3.480 (6)	158
C44—H44*B*⋯O8^i^	0.96	2.57	3.495 (8)	162
C45—H45*A*⋯O1*A* ^ii^	0.97	2.45	3.345 (8)	153
C45—H45*A*⋯O1^ii^	0.97	2.22	3.103 (9)	151

Symmetry codes: (i) 
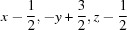
; (ii) 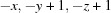; (iii) 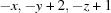.

**Fig. 1.**
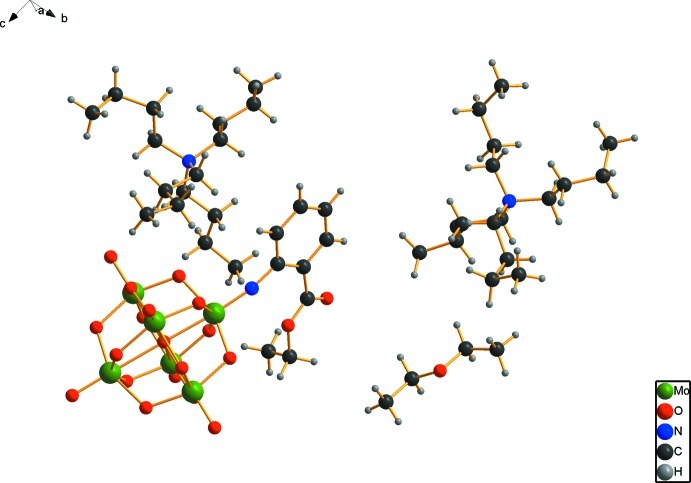
The asymmetric unit of the title compound. A single site for disordered groups has been retained. See the archived CIF for a complete view.

**Fig. 2.**
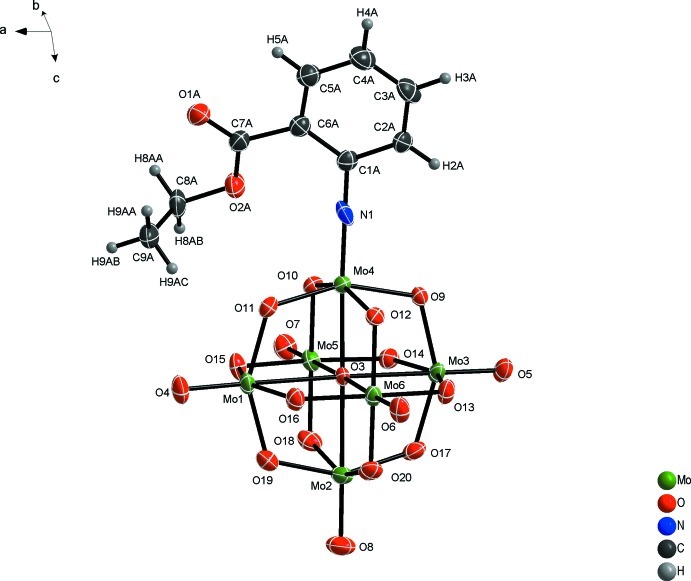
A view of the anion of the title compound, with displacement ellipsoids shown at the 50% probability level. One position of the disordered aryl group is shown.

**Fig. 3.**
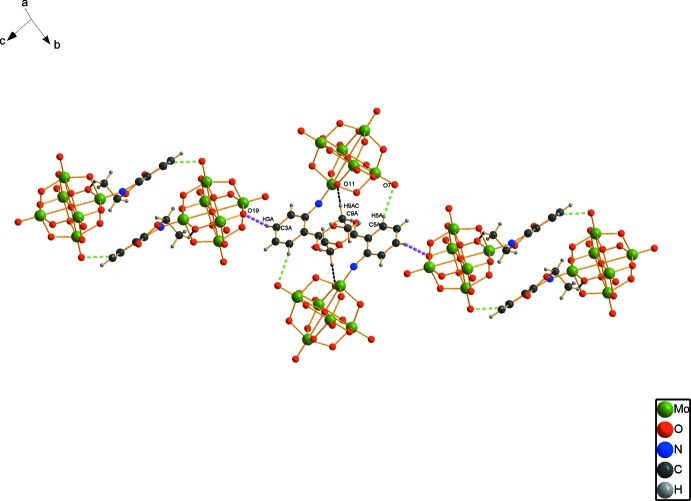
A view showing hydrogen bonds in the crystal.

## supplementary crystallographic information

### Comment

Polyoxometalates and their organoimido derivatives have gained interest owing to their important optical, electronic, magnetic, catalytic, medical, remarkable self-assembly properties and chemical reactivity (Hill, 1998; Li *et al.*, 2011). Up to date, a number of organoimido derivatives of hexamolybdate have been obtained *via* three types of reactions, which include reactions with phosphinimines (Du *et al.*, 1992), isocyanates (Mohs *et al.*, 1995), and aromatic amines (Clegg *et al.*, 1995). Also, based on the DCC dehydrating protocol, we have developed a new approach to synthesize such hybrid materials (Wu *et al.*, 2004).

Because the reaction of [α-Mo_8_O_26_]^4-^ with aromatic amines hydrochlorides can easily take place under much more milder conditions to selectively yield mono-functionalized organoimido derivatives of hexamolybdate, such new hybrids can now be synthesized more easily and conveniently in a more controlled fashion. As well known, the ester group is an important reactive functional group which can be applied in ester exchange, hydrolyzation, and aminating reactions in organic syntheses, thus it is necessary to develop new aromatic ester derivatives of hexamolybdate. Based on our previous work, we expanded aromatic ester amines as raw materials, and obtained more stable building blocks to construct novel POM-based organic-inorganic hybrids. Here, we report the synthesis and structural characterization of a new aromatic ester derivative of hexamolybdate.

The asymmetric unit of the title compound includes one [Mo_6_O_18_N(C_2_H_5_OOCC_6_H_4_)]^2-^ anion, two tetrabutylammonium cations, and a half ether molecule disordered by inversion (Fig. 1). Anions and cations are also disordered (see *Refinement* section). In the cluster anion one terminal oxo group is replaced by an alkylimido ligand (Fig. 2). The short Mo4≡N1 distance, 1.726 (4) Å, and approximately linear C1—N1≡Mo4 and C1A—N1≡Mo4 bond angles, 160.7 (5) and 167.6 (5)°, are typical of organoimido groups bonded at an octahedral *d*^0^ metal center, and are consistent with a significant degree of Mo≡N triple bond character (Karlin & Wigley, 2007; Li *et al.*, 2008). Another notable structural feature is that the Mo4—O3 distances between the Mo atom carrying the imido group and the central O atom within the cluster anion cage, 2.196 (3) Å, which is significantly shorter than the other Mo—O3 bond lengths (*ca*. 2.34 Å). This is consistent with the weak *trans*-influence of the imido group compared to the oxo group.

In the crystal, cations and anions form hydrogen bonds (Fig. 3), the donor groups being methylene groups in the cations and aromatic CH groups of the aryl ring, while acceptors groups are O atoms from the polyoxometalate cluster and the ester moieties.

### Experimental

A mixture of (Bu_4_N)_4_[α-Mo_8_O_26_] (1.0 mmol), DCC (2.7 mmol) and ethyl *o*-aminobenzoate hydrochloride (1.4 mmol) was refluxed in anhydrous acetonitrile (10 ml) for about 6 h. After dissolution, the mixture turned red, and a large amount of white precipitate formed, which was identified as dicyclohexyl urea. After cooling the suspension to room temperature, the white precipitate was removed by filtration, and acetonitrile was allowed to slowly evaporate. The product deposited from the filtrate as a red colloid-like solid. This red solid was washed with ethanol and Et_2_O several times, and the residue was dissolved in acetone. Single crystals used for X-ray diffraction were obtained by diffusion of ether into this solution. The product was obtained as orange crystals in moderate yield (50-60 %).

### Refinement

The structure is strongly disordered. All the arylester groups is disordered over two positions, for which occupancies were refined, converging to 0.559 (3) and 0.441 (3). In the N2-cation, C36 is disordered with C37. The N3-cation has three disordered butyl chains: C47—C48 is disordered with C49—C50; C54 is disordered with C55; and C57—C58—C59 is disordered with C60—C61—C62. Occupancies were restrained to be identical to those refined for the anion disorder. Finally, the occupancy for the ether molecule, which is placed on an inversion center, was fixed to 1/2. Bond lengths for C—C groups involving disordered parts of the cations were restrained with *DFIX* to suitable target values, as well as bond lengths in the ether molecule. A set of *SIMU* and *SAME* restraints were applied to the arylester, while *SIMU* and *DELU* restraints were used in the disordered parts of the cations. Finally, *SIMU* restraints were applied for the ether solvent (Sheldrick, 2008). All H atoms were placed in idealized positions, and refined as riding on their parent atoms, with C—H fixed to 0.93 (aromatic CH), 0.96 (methyl CH_3_) and 0.97 Å (methylene CH_2_). Isotropic displacement parameters for H atoms were calculated as *U*_iso_(H) = 1.2 *U*_eq_(parent C) for aromatic and methylene groups, and *U*_iso_(H) = 1. *U*_eq_(parent C) for methyl groups.

### Crystal data

**Table:**

(C_16_H_36_N)_2_[Mo_6_O_18_(C_9_H_9_NO_2_)]·0.5C_4_H_10_O	*F*(000) = 3124
*M**_r_* = 1548.79	*D*_x_ = 1.768 Mg m^−^^3^
Monoclinic, *P*2_1_/*n*	Mo *K*α radiation, λ = 0.71073 Å
Hall symbol: -P 2yn	Cell parameters from 9126 reflections
*a* = 16.3974 (16) Å	θ = 3.0–29.3°
*b* = 17.0854 (8) Å	µ = 1.33 mm^−^^1^
*c* = 21.687 (3) Å	*T* = 101 K
β = 106.709 (16)°	Prism, orange
*V* = 5819.1 (10) Å^3^	0.40 × 0.30 × 0.25 mm
*Z* = 4	

### Data collection

**Table:**

Agilent Xcalibur Eos Gemini diffractometer	11422 independent reflections
Radiation source: fine-focus sealed tube	8774 reflections with *I* > 2σ(*I*)
Graphite monochromator	*R*_int_ = 0.038
Detector resolution: 16.0971 pixels mm^-1^	θ_max_ = 26.0°, θ_min_ = 3.0°
ω scans	*h* = −18→20
Absorption correction: multi-scan (*CrysAlis PRO*; Agilent, 2011)	*k* = −21→20
*T*_min_ = 0.619, *T*_max_ = 0.733	*l* = −23→26
31709 measured reflections	

### Refinement

**Table:**

Refinement on *F*^2^	Primary atom site location: structure-invariant direct methods
Least-squares matrix: full	Secondary atom site location: difference Fourier map
*R*[*F*^2^ > 2σ(*F*^2^)] = 0.039	Hydrogen site location: inferred from neighbouring sites
*wR*(*F*^2^) = 0.088	H-atom parameters constrained
*S* = 1.05	*w* = 1/[σ^2^(*F*_o_^2^) + (0.0243*P*)^2^ + 15.7229*P*] where *P* = (*F*_o_^2^ + 2*F*_c_^2^)/3
11422 reflections	(Δ/σ)_max_ = 0.003
854 parameters	Δρ_max_ = 1.46 e Å^−^^3^
500 restraints	Δρ_min_ = −0.81 e Å^−^^3^
0 constraints	

### Fractional atomic coordinates and isotropic or equivalent isotropic displacement parameters (Å^2^)

**Table:**

	*x*	*y*	*z*	*U*_iso_*/*U*_eq_	Occ. (<1)
Mo1	0.13934 (2)	0.73051 (2)	0.69604 (2)	0.02405 (10)	
Mo2	0.02537 (3)	0.77620 (2)	0.79380 (2)	0.02889 (11)	
Mo3	−0.14888 (2)	0.71677 (2)	0.68630 (2)	0.02216 (10)	
Mo4	−0.03375 (2)	0.67183 (2)	0.592661 (19)	0.02048 (9)	
Mo5	0.01623 (2)	0.59741 (2)	0.73540 (2)	0.02414 (10)	
Mo6	−0.02612 (2)	0.84988 (2)	0.64686 (2)	0.02159 (10)	
O3	−0.00579 (17)	0.72276 (16)	0.68949 (15)	0.0201 (6)	
O4	0.24275 (19)	0.7419 (2)	0.69912 (18)	0.0357 (8)	
O5	−0.25062 (19)	0.70524 (18)	0.68807 (17)	0.0313 (8)	
O6	−0.04756 (19)	0.94123 (17)	0.61622 (17)	0.0315 (8)	
O7	0.0367 (2)	0.50668 (18)	0.76667 (17)	0.0331 (8)	
O8	0.0486 (2)	0.8132 (2)	0.86895 (18)	0.0442 (9)	
O9	−0.14729 (17)	0.67117 (16)	0.60821 (15)	0.0212 (7)	
O10	−0.00610 (18)	0.58029 (16)	0.64367 (15)	0.0222 (7)	
O11	0.08570 (18)	0.69854 (17)	0.60962 (15)	0.0241 (7)	
O12	−0.05685 (17)	0.78646 (16)	0.57403 (14)	0.0204 (6)	
O13	−0.14205 (18)	0.81707 (17)	0.65775 (17)	0.0287 (8)	
O14	−0.09814 (18)	0.61154 (17)	0.72434 (15)	0.0244 (7)	
O15	0.13247 (18)	0.63098 (18)	0.72674 (16)	0.0292 (8)	
O16	0.08876 (18)	0.83694 (17)	0.65953 (16)	0.0264 (7)	
O17	−0.0922 (2)	0.74992 (18)	0.77413 (16)	0.0309 (8)	
O18	0.04689 (19)	0.66369 (19)	0.80821 (15)	0.0306 (8)	
O19	0.13364 (19)	0.77597 (19)	0.77511 (16)	0.0322 (8)	
O20	−0.0036 (2)	0.86502 (17)	0.73992 (16)	0.0303 (8)	
N1	−0.0593 (2)	0.6384 (2)	0.51431 (19)	0.0244 (8)	
C1	−0.1064 (7)	0.6223 (9)	0.4506 (5)	0.024 (2)	0.441 (3)
C2	−0.1956 (6)	0.6319 (6)	0.4341 (5)	0.027 (2)	0.441 (3)
H2	−0.2212	0.6447	0.4659	0.032*	0.441 (3)
C3	−0.2451 (7)	0.6225 (6)	0.3711 (5)	0.032 (2)	0.441 (3)
H3	−0.3039	0.6280	0.3607	0.039*	0.441 (3)
C4	−0.2070 (7)	0.6049 (7)	0.3239 (6)	0.036 (2)	0.441 (3)
H4	−0.2405	0.5995	0.2815	0.043*	0.441 (3)
C5	−0.1199 (8)	0.5952 (8)	0.3386 (6)	0.036 (2)	0.441 (3)
H5	−0.0952	0.5841	0.3060	0.043*	0.441 (3)
C6	−0.0689 (7)	0.6019 (6)	0.4019 (5)	0.0275 (19)	0.441 (3)
C7	0.0246 (8)	0.5889 (7)	0.4151 (6)	0.0270 (19)	0.441 (3)
O1	0.0601 (5)	0.5984 (5)	0.3730 (4)	0.041 (2)	0.441 (3)
O2	0.0641 (6)	0.5669 (7)	0.4742 (5)	0.031 (2)	0.441 (3)
C8	0.1557 (6)	0.5532 (7)	0.4879 (5)	0.031 (2)	0.441 (3)
H8B	0.1798	0.5884	0.4626	0.038*	0.441 (3)
H8A	0.1663	0.4998	0.4772	0.038*	0.441 (3)
C9	0.1959 (11)	0.5681 (11)	0.5596 (8)	0.038 (4)	0.441 (3)
H9C	0.1929	0.6229	0.5684	0.057*	0.441 (3)
H9A	0.2543	0.5518	0.5716	0.057*	0.441 (3)
H9B	0.1656	0.5389	0.5839	0.057*	0.441 (3)
C1A	−0.0762 (6)	0.6282 (7)	0.4486 (4)	0.0255 (19)	0.559 (3)
C2A	−0.1593 (5)	0.6491 (5)	0.4117 (4)	0.0297 (17)	0.559 (3)
H2A	−0.1990	0.6663	0.4318	0.036*	0.559 (3)
C3A	−0.1807 (6)	0.6437 (5)	0.3455 (5)	0.0362 (19)	0.559 (3)
H3A	−0.2354	0.6572	0.3210	0.043*	0.559 (3)
C4A	−0.1225 (7)	0.6189 (6)	0.3155 (5)	0.039 (2)	0.559 (3)
H4A	−0.1375	0.6166	0.2708	0.046*	0.559 (3)
C5A	−0.0418 (5)	0.5972 (5)	0.3512 (4)	0.0327 (17)	0.559 (3)
H5A	−0.0032	0.5795	0.3302	0.039*	0.559 (3)
C6A	−0.0174 (7)	0.6012 (5)	0.4175 (4)	0.0268 (17)	0.559 (3)
C7A	0.0725 (8)	0.5762 (8)	0.4522 (6)	0.029 (2)	0.559 (3)
O1A	0.1254 (4)	0.5633 (4)	0.4235 (3)	0.0364 (15)	0.559 (3)
O2A	0.0876 (4)	0.5706 (4)	0.5147 (4)	0.0350 (15)	0.559 (3)
C8A	0.1737 (7)	0.5492 (9)	0.5516 (8)	0.036 (3)	0.559 (3)
H8AA	0.1934	0.5062	0.5305	0.043*	0.559 (3)
H8AB	0.1734	0.5318	0.5942	0.043*	0.559 (3)
C9A	0.2347 (6)	0.6189 (7)	0.5580 (5)	0.045 (3)	0.559 (3)
H9AA	0.2360	0.6353	0.5160	0.067*	0.559 (3)
H9AB	0.2908	0.6036	0.5829	0.067*	0.559 (3)
H9AC	0.2153	0.6614	0.5792	0.067*	0.559 (3)
N2	−0.1640 (2)	0.9199 (2)	0.39232 (18)	0.0223 (8)	
C21	−0.1699 (3)	0.8997 (3)	0.3232 (2)	0.0245 (10)	
H21A	−0.1548	0.8450	0.3216	0.029*	
H21B	−0.2288	0.9053	0.2977	0.029*	
C22	−0.1147 (3)	0.9479 (3)	0.2916 (2)	0.0333 (12)	
H22A	−0.0554	0.9437	0.3167	0.040*	
H22B	−0.1311	1.0025	0.2904	0.040*	
C23	−0.1248 (3)	0.9191 (3)	0.2237 (3)	0.0404 (13)	
H23A	−0.1082	0.8645	0.2255	0.048*	
H23B	−0.1845	0.9222	0.1995	0.048*	
C24	−0.0734 (4)	0.9641 (4)	0.1880 (3)	0.065 (2)	
H24A	−0.0814	0.9413	0.1462	0.097*	
H24B	−0.0142	0.9620	0.2117	0.097*	
H24C	−0.0919	1.0176	0.1833	0.097*	
C25	−0.0725 (3)	0.9137 (2)	0.4357 (2)	0.0228 (10)	
H25A	−0.0398	0.9562	0.4250	0.027*	
H25B	−0.0731	0.9215	0.4799	0.027*	
C26	−0.0259 (3)	0.8377 (3)	0.4326 (2)	0.0270 (10)	
H26A	−0.0272	0.8269	0.3884	0.032*	
H26B	−0.0536	0.7948	0.4479	0.032*	
C27	0.0666 (3)	0.8454 (3)	0.4747 (2)	0.0331 (12)	
H27A	0.0933	0.8889	0.4593	0.040*	
H27B	0.0670	0.8569	0.5186	0.040*	
C28	0.1178 (3)	0.7720 (3)	0.4743 (3)	0.0416 (13)	
H28A	0.1148	0.7585	0.4307	0.062*	
H28B	0.0951	0.7299	0.4936	0.062*	
H28C	0.1761	0.7809	0.4983	0.062*	
C29	−0.2222 (3)	0.8623 (3)	0.4138 (2)	0.0267 (10)	
H29A	−0.1959	0.8110	0.4180	0.032*	
H29B	−0.2755	0.8589	0.3798	0.032*	
C30	−0.2422 (3)	0.8808 (3)	0.4759 (2)	0.0262 (10)	
H30A	−0.1896	0.8864	0.5103	0.031*	
H30B	−0.2728	0.9300	0.4715	0.031*	
C31	−0.2960 (3)	0.8160 (3)	0.4928 (3)	0.0429 (14)	
H31A	−0.2609	0.7697	0.5053	0.052*	
H31B	−0.3418	0.8030	0.4546	0.052*	
C32	−0.3340 (3)	0.8376 (3)	0.5467 (3)	0.0417 (14)	
H32A	−0.3655	0.7939	0.5558	0.063*	
H32B	−0.2891	0.8510	0.5846	0.063*	
H32C	−0.3715	0.8816	0.5337	0.063*	
C33	−0.1902 (3)	1.0039 (2)	0.3993 (2)	0.0290 (11)	
H33A	−0.1511	1.0383	0.3863	0.035*	
H33B	−0.1839	1.0138	0.4445	0.035*	
C34	−0.2801 (3)	1.0255 (3)	0.3611 (3)	0.0368 (12)	
H34A	−0.2853	1.0257	0.3154	0.044*	
H34B	−0.3202	0.9877	0.3689	0.044*	
C35	−0.2989 (5)	1.1074 (4)	0.3830 (3)	0.067 (2)	
H35A	−0.3412	1.1328	0.3481	0.080*	0.559 (3)
H35B	−0.2473	1.1386	0.3928	0.080*	0.559 (3)
H35C	−0.2491	1.1391	0.3850	0.080*	0.441 (3)
H35D	−0.3014	1.1018	0.4269	0.080*	0.441 (3)
C36	−0.3310 (6)	1.1041 (6)	0.4418 (5)	0.043 (3)	0.559 (3)
H36A	−0.3734	1.0639	0.4362	0.065*	0.559 (3)
H36B	−0.2845	1.0928	0.4792	0.065*	0.559 (3)
H36C	−0.3557	1.1537	0.4474	0.065*	0.559 (3)
C37	−0.3721 (7)	1.1540 (7)	0.3496 (7)	0.050 (4)	0.441 (3)
H37A	−0.3679	1.1679	0.3078	0.075*	0.441 (3)
H37B	−0.4232	1.1243	0.3452	0.075*	0.441 (3)
H37C	−0.3738	1.2007	0.3739	0.075*	0.441 (3)
N3	−0.2747 (3)	0.4430 (2)	0.6884 (2)	0.0345 (10)	
C41	−0.2807 (3)	0.5100 (3)	0.6413 (2)	0.0328 (12)	
H41A	−0.2624	0.4910	0.6053	0.039*	
H41B	−0.2408	0.5504	0.6625	0.039*	
C42	−0.3682 (3)	0.5476 (4)	0.6148 (3)	0.0584 (18)	
H42A	−0.4108	0.5069	0.6011	0.070*	
H42B	−0.3817	0.5775	0.6486	0.070*	
C43	−0.3713 (3)	0.6013 (3)	0.5582 (3)	0.0485 (15)	
H43A	−0.3224	0.6359	0.5700	0.058*	
H43B	−0.4221	0.6334	0.5498	0.058*	
C44	−0.3717 (6)	0.5593 (5)	0.4983 (4)	0.086 (3)	
H44A	−0.3184	0.5328	0.5045	0.129*	
H44B	−0.3798	0.5962	0.4637	0.129*	
H44C	−0.4172	0.5218	0.4881	0.129*	
C45	−0.1809 (4)	0.4211 (3)	0.7129 (3)	0.0507 (17)	
H45A	−0.1612	0.4071	0.6762	0.061*	
H45B	−0.1492	0.4669	0.7327	0.061*	
C46	−0.1597 (5)	0.3539 (4)	0.7613 (4)	0.082 (3)	
H46A	−0.1916	0.3077	0.7423	0.099*	0.559 (3)
H46B	−0.1768	0.3679	0.7991	0.099*	0.559 (3)
H46C	−0.2045	0.3148	0.7502	0.099*	0.441 (3)
H46D	−0.1553	0.3733	0.8042	0.099*	0.441 (3)
C47	−0.0640 (9)	0.3350 (8)	0.7814 (7)	0.053 (3)	0.559 (3)
H47A	−0.0502	0.3157	0.7435	0.064*	0.559 (3)
H47B	−0.0337	0.3840	0.7933	0.064*	0.559 (3)
C48	−0.0274 (8)	0.2766 (6)	0.8361 (6)	0.051 (3)	0.559 (3)
H48A	0.0267	0.2579	0.8335	0.077*	0.559 (3)
H48B	−0.0658	0.2333	0.8323	0.077*	0.559 (3)
H48C	−0.0202	0.3021	0.8768	0.077*	0.559 (3)
C49	−0.0756 (12)	0.3176 (11)	0.7597 (11)	0.068 (5)	0.441 (3)
H49A	−0.0773	0.3048	0.7158	0.082*	0.441 (3)
H49B	−0.0294	0.3542	0.7766	0.082*	0.441 (3)
C50	−0.0621 (9)	0.2445 (8)	0.8004 (8)	0.050 (4)	0.441 (3)
H50A	−0.0970	0.2031	0.7768	0.075*	0.441 (3)
H50B	−0.0033	0.2294	0.8112	0.075*	0.441 (3)
H50C	−0.0775	0.2546	0.8392	0.075*	0.441 (3)
C51	−0.3090 (3)	0.4661 (3)	0.7443 (2)	0.0324 (12)	
H51A	−0.3049	0.4211	0.7724	0.039*	
H51B	−0.3689	0.4791	0.7273	0.039*	
C52	−0.2644 (4)	0.5333 (4)	0.7835 (3)	0.0555 (17)	
H52A	−0.2048	0.5200	0.8020	0.067*	
H52B	−0.2671	0.5782	0.7555	0.067*	
C53	−0.3021 (4)	0.5557 (4)	0.8372 (3)	0.0491 (15)	
H53A	−0.3297	0.5111	0.8504	0.059*	0.559 (3)
H53B	−0.2585	0.5753	0.8743	0.059*	0.559 (3)
H53C	−0.3637	0.5552	0.8200	0.059*	0.441 (3)
H53D	−0.2868	0.5155	0.8701	0.059*	0.441 (3)
C54	−0.3657 (9)	0.6184 (8)	0.8085 (7)	0.083 (5)	0.559 (3)
H54A	−0.4148	0.5952	0.7787	0.125*	0.559 (3)
H54B	−0.3405	0.6555	0.7862	0.125*	0.559 (3)
H54C	−0.3825	0.6445	0.8421	0.125*	0.559 (3)
C55	−0.2766 (8)	0.6321 (7)	0.8683 (7)	0.047 (3)	0.441 (3)
H55A	−0.2969	0.6732	0.8375	0.071*	0.441 (3)
H55B	−0.2157	0.6346	0.8842	0.071*	0.441 (3)
H55C	−0.3007	0.6383	0.9035	0.071*	0.441 (3)
C56	−0.3273 (4)	0.3745 (3)	0.6559 (3)	0.0604 (16)	
H56A	−0.3803	0.3932	0.6268	0.072*	0.559 (3)
H56B	−0.3407	0.3414	0.6880	0.072*	0.559 (3)
H56C	−0.3063	0.3286	0.6819	0.072*	0.441 (3)
H56D	−0.3851	0.3833	0.6575	0.072*	0.441 (3)
C57	−0.3322 (8)	0.3535 (7)	0.5855 (6)	0.040 (2)	0.441 (3)
H57A	−0.2786	0.3301	0.5848	0.048*	0.441 (3)
H57B	−0.3394	0.4014	0.5605	0.048*	0.441 (3)
C58	−0.4051 (16)	0.2968 (14)	0.5529 (9)	0.040 (3)	0.441 (3)
H58A	−0.4044	0.2524	0.5809	0.048*	0.441 (3)
H58B	−0.4594	0.3233	0.5456	0.048*	0.441 (3)
C59	−0.3947 (9)	0.2684 (9)	0.4888 (7)	0.052 (3)	0.441 (3)
H59A	−0.3414	0.2414	0.4962	0.078*	0.441 (3)
H59B	−0.3956	0.3125	0.4611	0.078*	0.441 (3)
H59C	−0.4405	0.2336	0.4686	0.078*	0.441 (3)
C60	−0.2759 (6)	0.3243 (6)	0.6160 (5)	0.0432 (19)	0.559 (3)
H60A	−0.2594	0.3574	0.5853	0.052*	0.559 (3)
H60B	−0.2249	0.3017	0.6450	0.052*	0.559 (3)
C61	−0.3362 (6)	0.2602 (6)	0.5814 (5)	0.041 (2)	0.559 (3)
H61A	−0.3051	0.2243	0.5618	0.050*	0.559 (3)
H61B	−0.3558	0.2310	0.6127	0.050*	0.559 (3)
C62	−0.4127 (12)	0.2906 (12)	0.5299 (7)	0.053 (4)	0.559 (3)
H62A	−0.3939	0.3234	0.5006	0.079*	0.559 (3)
H62B	−0.4445	0.2473	0.5067	0.079*	0.559 (3)
H62C	−0.4481	0.3204	0.5495	0.079*	0.559 (3)
C101	0.0391 (19)	0.4428 (14)	1.1046 (8)	0.072 (6)	0.50
H10H	−0.0166	0.4232	1.1023	0.107*	0.50
H10I	0.0814	0.4093	1.1318	0.107*	0.50
H10J	0.0452	0.4948	1.1222	0.107*	0.50
C102	0.0505 (8)	0.4445 (8)	1.0375 (6)	0.059 (3)	0.50
H10F	0.0373	0.3938	1.0171	0.071*	0.50
H10G	0.1087	0.4579	1.0397	0.071*	0.50
O103	−0.008 (2)	0.503 (2)	1.0017 (11)	0.043 (3)	0.50
C104	0.0004 (8)	0.5075 (7)	0.9368 (6)	0.051 (3)	0.50
H10D	−0.0249	0.4620	0.9117	0.061*	0.50
H10E	0.0595	0.5116	0.9372	0.061*	0.50
C105	−0.0486 (16)	0.5817 (10)	0.9103 (10)	0.064 (5)	0.50
H10A	−0.0479	0.5895	0.8666	0.096*	0.50
H10B	−0.1064	0.5767	0.9115	0.096*	0.50
H10C	−0.0224	0.6257	0.9360	0.096*	0.50

### Atomic displacement parameters (Å^2^)

**Table:**

	*U*^11^	*U*^22^	*U*^33^	*U*^12^	*U*^13^	*U*^23^
Mo1	0.01366 (18)	0.0267 (2)	0.0309 (2)	−0.00060 (15)	0.00505 (16)	−0.00190 (18)
Mo2	0.0269 (2)	0.0363 (2)	0.0227 (2)	−0.00515 (18)	0.00589 (17)	−0.00936 (19)
Mo3	0.01683 (19)	0.02216 (18)	0.0299 (2)	−0.00188 (15)	0.01057 (16)	−0.00479 (17)
Mo4	0.01943 (19)	0.02174 (18)	0.0208 (2)	−0.00087 (15)	0.00670 (15)	−0.00375 (16)
Mo5	0.0206 (2)	0.02525 (19)	0.0251 (2)	0.00071 (16)	0.00420 (16)	0.00308 (17)
Mo6	0.01640 (18)	0.01828 (18)	0.0296 (2)	−0.00127 (14)	0.00589 (16)	−0.00219 (16)
O3	0.0121 (13)	0.0237 (14)	0.0246 (17)	−0.0008 (11)	0.0055 (12)	−0.0037 (13)
O4	0.0163 (16)	0.0409 (19)	0.048 (2)	−0.0009 (14)	0.0070 (15)	0.0014 (17)
O5	0.0228 (16)	0.0302 (17)	0.045 (2)	−0.0034 (14)	0.0166 (15)	−0.0086 (16)
O6	0.0235 (16)	0.0216 (15)	0.049 (2)	−0.0024 (13)	0.0092 (15)	−0.0015 (15)
O7	0.0320 (18)	0.0315 (17)	0.034 (2)	0.0018 (15)	0.0055 (15)	0.0074 (16)
O8	0.046 (2)	0.056 (2)	0.030 (2)	−0.0121 (19)	0.0106 (17)	−0.0178 (19)
O9	0.0157 (14)	0.0222 (15)	0.0247 (18)	−0.0019 (12)	0.0045 (12)	−0.0026 (13)
O10	0.0241 (16)	0.0201 (14)	0.0230 (18)	0.0013 (12)	0.0078 (13)	−0.0022 (13)
O11	0.0233 (16)	0.0266 (15)	0.0260 (19)	0.0026 (13)	0.0129 (14)	−0.0007 (14)
O12	0.0209 (15)	0.0211 (14)	0.0189 (17)	0.0007 (12)	0.0052 (13)	0.0009 (13)
O13	0.0201 (16)	0.0210 (15)	0.047 (2)	0.0021 (13)	0.0123 (15)	−0.0031 (15)
O14	0.0246 (16)	0.0273 (16)	0.0240 (18)	−0.0028 (13)	0.0111 (14)	−0.0010 (14)
O15	0.0180 (15)	0.0307 (17)	0.036 (2)	0.0041 (13)	0.0030 (14)	0.0093 (15)
O16	0.0200 (16)	0.0252 (16)	0.035 (2)	−0.0034 (13)	0.0095 (14)	−0.0021 (14)
O17	0.0344 (18)	0.0331 (17)	0.031 (2)	−0.0032 (15)	0.0185 (15)	−0.0110 (15)
O18	0.0316 (18)	0.0392 (18)	0.0192 (19)	−0.0083 (15)	0.0047 (14)	0.0013 (15)
O19	0.0249 (17)	0.0387 (18)	0.029 (2)	−0.0030 (14)	0.0016 (14)	−0.0070 (16)
O20	0.0321 (18)	0.0244 (16)	0.036 (2)	−0.0074 (14)	0.0124 (15)	−0.0127 (15)
N1	0.0263 (19)	0.0233 (18)	0.025 (2)	0.0017 (15)	0.0090 (16)	−0.0007 (16)
C1	0.028 (4)	0.023 (4)	0.024 (4)	0.002 (4)	0.012 (4)	−0.001 (3)
C2	0.029 (4)	0.028 (4)	0.025 (5)	0.006 (4)	0.011 (4)	0.002 (4)
C3	0.029 (4)	0.032 (4)	0.030 (4)	0.013 (4)	0.001 (4)	0.001 (4)
C4	0.039 (4)	0.040 (5)	0.026 (5)	0.007 (4)	0.004 (4)	0.007 (4)
C5	0.039 (4)	0.038 (4)	0.029 (5)	0.005 (4)	0.006 (4)	0.003 (4)
C6	0.029 (4)	0.028 (3)	0.028 (4)	0.002 (4)	0.012 (3)	−0.001 (3)
C7	0.027 (4)	0.028 (4)	0.030 (4)	−0.002 (4)	0.015 (4)	−0.005 (3)
O1	0.039 (4)	0.053 (5)	0.037 (5)	−0.007 (4)	0.021 (4)	−0.002 (4)
O2	0.028 (3)	0.037 (3)	0.030 (4)	0.001 (3)	0.010 (4)	−0.004 (4)
C8	0.022 (4)	0.040 (4)	0.036 (4)	0.004 (3)	0.013 (3)	−0.001 (4)
C9	0.016 (7)	0.046 (8)	0.043 (7)	0.001 (6)	−0.006 (6)	−0.008 (7)
C1A	0.029 (4)	0.021 (3)	0.026 (4)	0.004 (4)	0.007 (3)	0.001 (3)
C2A	0.027 (4)	0.028 (3)	0.032 (4)	0.008 (3)	0.005 (3)	−0.004 (3)
C3A	0.033 (4)	0.034 (4)	0.032 (4)	0.005 (3)	−0.006 (3)	0.004 (3)
C4A	0.039 (4)	0.043 (4)	0.028 (5)	0.003 (4)	0.000 (4)	0.001 (4)
C5A	0.035 (3)	0.033 (3)	0.031 (4)	0.002 (3)	0.009 (3)	−0.002 (3)
C6A	0.028 (4)	0.025 (3)	0.028 (3)	0.001 (3)	0.009 (3)	0.000 (3)
C7A	0.027 (4)	0.028 (3)	0.033 (5)	0.002 (3)	0.010 (4)	−0.005 (4)
O1A	0.031 (3)	0.048 (3)	0.034 (4)	−0.005 (3)	0.015 (3)	−0.010 (3)
O2A	0.026 (3)	0.044 (3)	0.034 (4)	0.008 (3)	0.006 (3)	0.003 (3)
C8A	0.019 (5)	0.045 (5)	0.043 (5)	0.009 (4)	0.006 (4)	0.004 (5)
C9A	0.027 (5)	0.068 (7)	0.036 (6)	0.015 (5)	0.003 (4)	−0.007 (5)
N2	0.0163 (18)	0.0225 (18)	0.026 (2)	−0.0048 (14)	0.0020 (16)	0.0008 (16)
C21	0.022 (2)	0.026 (2)	0.022 (3)	−0.0051 (19)	0.0007 (19)	0.000 (2)
C22	0.032 (3)	0.040 (3)	0.025 (3)	−0.013 (2)	0.004 (2)	0.001 (2)
C23	0.040 (3)	0.049 (3)	0.031 (3)	−0.019 (3)	0.008 (2)	−0.009 (3)
C24	0.086 (5)	0.080 (5)	0.030 (4)	−0.051 (4)	0.019 (3)	−0.013 (3)
C25	0.019 (2)	0.029 (2)	0.018 (2)	−0.0077 (18)	0.0014 (18)	−0.0025 (19)
C26	0.021 (2)	0.037 (3)	0.021 (3)	−0.003 (2)	0.0034 (19)	−0.001 (2)
C27	0.023 (2)	0.048 (3)	0.029 (3)	−0.005 (2)	0.008 (2)	0.000 (2)
C28	0.028 (3)	0.058 (3)	0.038 (3)	0.003 (2)	0.008 (2)	0.009 (3)
C29	0.020 (2)	0.026 (2)	0.031 (3)	−0.0051 (18)	0.003 (2)	0.002 (2)
C30	0.017 (2)	0.031 (2)	0.027 (3)	0.0011 (19)	0.0009 (19)	0.004 (2)
C31	0.043 (3)	0.038 (3)	0.054 (4)	−0.005 (2)	0.024 (3)	0.008 (3)
C32	0.031 (3)	0.051 (3)	0.048 (4)	0.006 (2)	0.018 (3)	0.015 (3)
C33	0.035 (3)	0.019 (2)	0.033 (3)	−0.005 (2)	0.010 (2)	0.000 (2)
C34	0.042 (3)	0.035 (3)	0.034 (3)	0.013 (2)	0.013 (2)	0.011 (2)
C35	0.101 (6)	0.046 (4)	0.066 (5)	0.033 (4)	0.045 (4)	0.024 (3)
C36	0.036 (5)	0.041 (5)	0.051 (7)	0.002 (4)	0.010 (5)	−0.005 (5)
C37	0.037 (7)	0.048 (7)	0.064 (10)	0.002 (6)	0.013 (7)	−0.004 (7)
N3	0.045 (3)	0.024 (2)	0.044 (3)	−0.0119 (18)	0.027 (2)	−0.0028 (19)
C41	0.035 (3)	0.037 (3)	0.029 (3)	−0.010 (2)	0.014 (2)	0.002 (2)
C42	0.033 (3)	0.091 (5)	0.052 (4)	−0.005 (3)	0.015 (3)	0.009 (4)
C43	0.032 (3)	0.056 (4)	0.052 (4)	−0.001 (3)	0.002 (3)	0.006 (3)
C44	0.123 (7)	0.082 (5)	0.055 (5)	0.025 (5)	0.031 (5)	0.016 (4)
C45	0.068 (4)	0.033 (3)	0.071 (5)	0.018 (3)	0.053 (4)	0.020 (3)
C46	0.098 (4)	0.062 (4)	0.120 (6)	0.051 (4)	0.084 (5)	0.059 (4)
C47	0.084 (4)	0.023 (5)	0.074 (9)	0.016 (5)	0.056 (6)	0.014 (5)
C48	0.057 (6)	0.041 (6)	0.056 (8)	−0.017 (4)	0.020 (5)	0.009 (5)
C49	0.084 (8)	0.050 (9)	0.097 (12)	0.033 (7)	0.068 (8)	0.034 (8)
C50	0.038 (7)	0.039 (7)	0.073 (11)	0.010 (5)	0.018 (7)	0.017 (6)
C51	0.034 (3)	0.035 (3)	0.036 (3)	−0.002 (2)	0.023 (2)	0.002 (2)
C52	0.060 (4)	0.058 (4)	0.062 (5)	−0.020 (3)	0.039 (3)	−0.018 (3)
C53	0.052 (4)	0.059 (4)	0.045 (4)	−0.012 (3)	0.026 (3)	−0.007 (3)
C54	0.086 (10)	0.090 (10)	0.097 (12)	0.017 (8)	0.064 (9)	−0.012 (9)
C55	0.051 (8)	0.050 (7)	0.046 (9)	0.007 (6)	0.022 (7)	−0.011 (7)
C56	0.090 (4)	0.048 (3)	0.063 (4)	−0.035 (3)	0.053 (3)	−0.021 (3)
C57	0.048 (5)	0.030 (4)	0.048 (4)	−0.012 (4)	0.026 (4)	−0.007 (4)
C58	0.044 (6)	0.038 (5)	0.045 (7)	−0.006 (5)	0.024 (6)	−0.013 (6)
C59	0.052 (7)	0.055 (7)	0.049 (8)	−0.010 (6)	0.017 (6)	−0.018 (7)
C60	0.044 (4)	0.038 (4)	0.053 (5)	−0.017 (3)	0.023 (4)	−0.006 (4)
C61	0.041 (4)	0.039 (4)	0.050 (5)	−0.006 (4)	0.022 (4)	−0.009 (4)
C62	0.047 (6)	0.061 (7)	0.055 (9)	−0.007 (5)	0.023 (8)	−0.021 (8)
C101	0.077 (10)	0.080 (12)	0.062 (12)	0.017 (10)	0.028 (9)	0.020 (10)
C102	0.050 (6)	0.061 (6)	0.058 (7)	0.006 (5)	0.004 (5)	0.008 (6)
O103	0.044 (8)	0.046 (5)	0.044 (4)	0.007 (5)	0.021 (4)	0.002 (4)
C104	0.051 (6)	0.065 (6)	0.039 (6)	−0.004 (5)	0.016 (5)	0.010 (5)
C105	0.069 (11)	0.077 (12)	0.043 (10)	−0.002 (9)	0.011 (8)	0.011 (9)

### Geometric parameters (Å, º)

**Table:**

Mo1—O3	2.347 (3)	C32—H32A	0.9600
Mo1—O4	1.689 (3)	C32—H32B	0.9600
Mo1—O11	1.907 (3)	C32—H32C	0.9600
Mo1—O15	1.841 (3)	C33—C34	1.514 (7)
Mo1—O16	2.060 (3)	C33—H33A	0.9700
Mo1—O19	1.909 (3)	C33—H33B	0.9700
Mo2—O3	2.355 (3)	C34—C35	1.538 (7)
Mo2—O8	1.686 (4)	C34—H34A	0.9700
Mo2—O17	1.904 (3)	C34—H34B	0.9700
Mo2—O18	1.963 (3)	C35—C36	1.515 (12)
Mo2—O19	1.931 (3)	C35—C37	1.449 (8)
Mo2—O20	1.891 (3)	C35—H35A	0.9700
Mo3—O3	2.330 (3)	C35—H35B	0.9700
Mo3—O5	1.691 (3)	C35—H35C	0.9700
Mo3—O9	1.871 (3)	C35—H35D	0.9700
Mo3—O13	1.836 (3)	C36—H36A	0.9600
Mo3—O14	2.052 (3)	C36—H36B	0.9600
Mo3—O17	1.948 (3)	C36—H36C	0.9600
Mo4—N1	1.726 (4)	C37—H37A	0.9600
Mo4—O3	2.196 (3)	C37—H37B	0.9600
Mo4—O9	1.985 (3)	C37—H37C	0.9600
Mo4—O10	1.894 (3)	N3—C41	1.519 (6)
Mo4—O11	1.941 (3)	N3—C45	1.523 (7)
Mo4—O12	2.013 (3)	N3—C51	1.529 (6)
Mo5—O3	2.346 (3)	N3—C56	1.502 (6)
Mo5—O7	1.687 (3)	C41—C42	1.525 (7)
Mo5—O10	1.939 (3)	C41—H41A	0.9700
Mo5—O14	1.837 (3)	C41—H41B	0.9700
Mo5—O15	2.050 (3)	C42—H42A	0.9700
Mo5—O18	1.890 (3)	C42—H42B	0.9700
Mo6—O3	2.346 (3)	C43—C42	1.520 (8)
Mo6—O6	1.693 (3)	C43—C44	1.481 (9)
Mo6—O12	1.862 (3)	C43—H43A	0.9700
Mo6—O13	2.059 (3)	C43—H43B	0.9700
Mo6—O16	1.837 (3)	C44—H44A	0.9600
Mo6—O20	1.961 (3)	C44—H44B	0.9600
N1—C1	1.403 (10)	C44—H44C	0.9600
C1—C2	1.411 (12)	C45—C46	1.527 (7)
C1—C6	1.409 (13)	C45—H45A	0.9700
C2—C3	1.383 (12)	C45—H45B	0.9700
C2—H2	0.9300	C46—C49	1.521 (16)
C3—C4	1.378 (14)	C46—H46A	0.9700
C3—H3	0.9300	C46—H46B	0.9700
C4—C5	1.381 (16)	C46—H46C	0.9700
C4—H4	0.9300	C46—H46D	0.9700
C5—C6	1.392 (14)	C47—C46	1.537 (14)
C5—H5	0.9300	C47—C48	1.535 (13)
C6—C7	1.494 (13)	C47—H47A	0.9700
C7—O1	1.227 (11)	C47—H47B	0.9700
C7—O2	1.312 (15)	C48—H48A	0.9600
O2—C8	1.464 (12)	C48—H48B	0.9600
C8—C9	1.526 (16)	C48—H48C	0.9600
C8—H8A	0.9700	C49—H49A	0.9700
C8—H8B	0.9700	C49—H49B	0.9700
C9—H9C	0.9600	C50—C49	1.509 (16)
C9—H9A	0.9600	C50—H50A	0.9600
C9—H9B	0.9600	C50—H50B	0.9600
N1—C1A	1.383 (9)	C50—H50C	0.9600
C1A—C2A	1.412 (11)	C51—C52	1.490 (7)
C1A—C6A	1.404 (12)	C51—H51A	0.9700
C2A—C3A	1.379 (11)	C51—H51B	0.9700
C2A—H2A	0.9300	C52—H52A	0.9700
C3A—C4A	1.368 (13)	C52—H52B	0.9700
C3A—H3A	0.9300	C53—C52	1.517 (8)
C4A—C5A	1.377 (13)	C53—C54	1.500 (14)
C4A—H4A	0.9300	C53—C55	1.474 (13)
C5A—C6A	1.380 (11)	C53—H53A	0.9700
C5A—H5A	0.9300	C53—H53B	0.9700
C6A—C7A	1.512 (13)	C53—H53C	0.9700
C7A—O1A	1.224 (11)	C53—H53D	0.9700
C7A—O2A	1.308 (13)	C54—H54A	0.9600
O2A—C8A	1.455 (13)	C54—H54B	0.9600
C8A—C9A	1.535 (13)	C54—H54C	0.9600
C8A—H8AA	0.9700	C55—H55A	0.9600
C8A—H8AB	0.9700	C55—H55B	0.9600
C9A—H9AA	0.9600	C55—H55C	0.9600
C9A—H9AB	0.9600	C56—C57	1.547 (14)
C9A—H9AC	0.9600	C56—C60	1.616 (11)
N2—C21	1.514 (6)	C56—H56A	0.9700
N2—C25	1.530 (5)	C56—H56B	0.9700
N2—C29	1.533 (5)	C56—H56C	0.9700
N2—C33	1.517 (5)	C56—H56D	0.9700
C21—C22	1.525 (6)	C57—C58	1.54 (3)
C21—H21A	0.9700	C57—H57A	0.9700
C21—H21B	0.9700	C57—H57B	0.9700
C22—C23	1.516 (7)	C58—H58A	0.9700
C22—H22A	0.9700	C58—H58B	0.9700
C22—H22B	0.9700	C59—C58	1.53 (2)
C23—C24	1.509 (7)	C59—H59A	0.9600
C23—H23A	0.9700	C59—H59B	0.9600
C23—H23B	0.9700	C59—H59C	0.9600
C24—H24A	0.9600	C60—C61	1.522 (13)
C24—H24B	0.9600	C60—H60A	0.9700
C24—H24C	0.9600	C60—H60B	0.9700
C25—C26	1.517 (6)	C61—H61A	0.9700
C25—H25A	0.9700	C61—H61B	0.9700
C25—H25B	0.9700	C62—C61	1.51 (2)
C26—C27	1.535 (6)	C62—H62A	0.9600
C26—H26A	0.9700	C62—H62B	0.9600
C26—H26B	0.9700	C62—H62C	0.9600
C27—H27A	0.9700	C101—H10H	0.9600
C27—H27B	0.9700	C101—H10I	0.9600
C28—C27	1.510 (7)	C101—H10J	0.9600
C28—H28A	0.9600	C102—C101	1.520 (10)
C28—H28B	0.9600	C102—H10F	0.9700
C28—H28C	0.9600	C102—H10G	0.9700
C29—C30	1.509 (6)	O103—C102	1.447 (18)
C29—H29A	0.9700	O103—C104	1.452 (19)
C29—H29B	0.9700	C104—H10D	0.9700
C30—C31	1.524 (6)	C104—H10E	0.9700
C30—H30A	0.9700	C105—C104	1.522 (9)
C30—H30B	0.9700	C105—H10A	0.9600
C31—H31A	0.9700	C105—H10B	0.9600
C31—H31B	0.9700	C105—H10C	0.9600
C32—C31	1.520 (7)		
			
O4—Mo1—O3	176.42 (14)	C28—C27—H27A	109.1
O4—Mo1—O11	104.24 (15)	C28—C27—H27B	109.1
O4—Mo1—O15	104.91 (15)	H27A—C27—H27B	107.8
O4—Mo1—O16	101.65 (14)	C27—C28—H28A	109.5
O4—Mo1—O19	103.03 (16)	C27—C28—H28B	109.5
O11—Mo1—O3	75.59 (11)	C27—C28—H28C	109.5
O11—Mo1—O16	81.61 (12)	H28A—C28—H28B	109.5
O11—Mo1—O19	150.63 (13)	H28B—C28—H28C	109.5
O15—Mo1—O3	78.66 (11)	H28A—C28—H28C	109.5
O15—Mo1—O11	91.83 (14)	C30—C29—N2	116.8 (4)
O15—Mo1—O16	153.44 (12)	C30—C29—H29B	108.1
O15—Mo1—O19	91.55 (15)	N2—C29—H29A	108.1
O16—Mo1—O3	74.77 (11)	N2—C29—H29B	108.1
O19—Mo1—O3	76.51 (12)	C30—C29—H29A	108.1
O19—Mo1—O16	82.41 (13)	H29B—C29—H29A	107.3
O8—Mo2—O3	179.10 (16)	C29—C30—C31	110.6 (4)
O8—Mo2—O17	103.75 (16)	C29—C30—H30A	109.5
O8—Mo2—O18	103.47 (17)	C29—C30—H30B	109.5
O8—Mo2—O19	103.76 (16)	C31—C30—H30A	109.5
O8—Mo2—O20	104.08 (17)	C31—C30—H30B	109.5
O17—Mo2—O3	76.54 (12)	H30B—C30—H30A	108.1
O17—Mo2—O18	85.92 (13)	C32—C31—C30	113.7 (4)
O17—Mo2—O19	152.23 (14)	C30—C31—H31A	108.8
O18—Mo2—O3	75.68 (12)	C30—C31—H31B	108.8
O19—Mo2—O3	75.89 (12)	C32—C31—H31A	108.8
O19—Mo2—O18	84.11 (13)	C32—C31—H31B	108.8
O20—Mo2—O3	76.76 (12)	H31B—C31—H31A	107.7
O20—Mo2—O17	89.59 (14)	C31—C32—H32A	109.5
O20—Mo2—O18	152.37 (14)	C31—C32—H32B	109.5
O20—Mo2—O19	87.39 (14)	C31—C32—H32C	109.5
O5—Mo3—O3	174.93 (14)	H32B—C32—H32A	109.5
O5—Mo3—O9	104.04 (14)	H32C—C32—H32A	109.5
O5—Mo3—O13	105.81 (14)	H32C—C32—H32B	109.5
O5—Mo3—O14	100.22 (14)	C34—C33—N2	115.8 (4)
O5—Mo3—O17	102.03 (15)	C34—C33—H33B	108.3
O9—Mo3—O3	76.66 (11)	N2—C33—H33A	108.3
O9—Mo3—O14	83.66 (12)	N2—C33—H33B	108.3
O9—Mo3—O17	151.53 (12)	C34—C33—H33A	108.3
O13—Mo3—O3	79.08 (11)	H33B—C33—H33A	107.4
O13—Mo3—O9	93.74 (14)	C33—C34—C35	107.6 (5)
O13—Mo3—O14	153.66 (12)	C33—C34—H34A	110.2
O13—Mo3—O17	90.00 (14)	C33—C34—H34B	110.2
O14—Mo3—O3	74.81 (11)	C35—C34—H34A	110.2
O17—Mo3—O3	76.38 (12)	C35—C34—H34B	110.2
O17—Mo3—O14	80.64 (13)	H34B—C34—H34A	108.5
N1—Mo4—O3	175.66 (14)	C34—C35—H35A	109.2
N1—Mo4—O9	101.01 (15)	C34—C35—H35B	109.2
N1—Mo4—O10	104.57 (15)	C34—C35—H35C	106.4
N1—Mo4—O11	102.16 (15)	C34—C35—H35D	106.4
N1—Mo4—O12	98.37 (15)	C36—C35—C34	112.0 (6)
O9—Mo4—O3	77.76 (11)	C36—C35—H35A	109.2
O9—Mo4—O12	84.73 (11)	C36—C35—H35B	109.2
O10—Mo4—O3	79.61 (12)	C37—C35—C34	123.8 (8)
O10—Mo4—O9	88.56 (12)	C37—C35—H35B	110.6
O10—Mo4—O11	91.39 (12)	C37—C35—H35C	106.4
O10—Mo4—O12	156.94 (12)	C37—C35—H35D	106.4
O11—Mo4—O3	78.68 (11)	H35B—C35—H35A	107.9
O11—Mo4—O9	156.06 (12)	H35D—C35—H35C	106.4
O11—Mo4—O12	86.02 (12)	C35—C36—H36A	109.5
O12—Mo4—O3	77.41 (11)	C35—C36—H36B	109.5
O7—Mo5—O3	176.81 (13)	C35—C36—H36C	109.5
O7—Mo5—O10	103.36 (15)	C35—C37—H37A	109.5
O7—Mo5—O14	104.52 (14)	C35—C37—H37B	109.5
O7—Mo5—O15	102.21 (14)	C35—C37—H37C	109.5
O7—Mo5—O18	103.90 (16)	H37A—C37—H37B	109.5
O10—Mo5—O3	75.03 (11)	H37A—C37—H37C	109.5
O10—Mo5—O15	81.63 (13)	H37C—C37—H37B	109.5
O14—Mo5—O3	78.33 (11)	C56—N3—C41	110.8 (4)
O14—Mo5—O10	90.08 (13)	C56—N3—C45	111.0 (4)
O14—Mo5—O15	153.18 (13)	C41—N3—C45	106.5 (3)
O14—Mo5—O18	92.67 (14)	C56—N3—C51	106.3 (4)
O15—Mo5—O3	74.89 (10)	C41—N3—C51	111.8 (4)
O18—Mo5—O3	77.23 (12)	C45—N3—C51	110.6 (4)
O18—Mo5—O10	150.94 (13)	N3—C41—C42	116.3 (4)
O18—Mo5—O15	82.99 (14)	N3—C41—H41A	108.2
O6—Mo6—O3	176.21 (12)	C42—C41—H41A	108.2
O6—Mo6—O12	103.21 (15)	N3—C41—H41B	108.2
O6—Mo6—O13	101.63 (13)	C42—C41—H41B	108.2
O6—Mo6—O16	104.94 (14)	H41A—C41—H41B	107.4
O6—Mo6—O20	103.81 (15)	C43—C42—C41	111.6 (4)
O12—Mo6—O3	76.62 (11)	C43—C42—H42A	109.3
O12—Mo6—O13	84.56 (13)	C41—C42—H42A	109.3
O12—Mo6—O20	151.02 (12)	C43—C42—H42B	109.3
O13—Mo6—O3	74.58 (11)	C41—C42—H42B	109.3
O16—Mo6—O3	78.83 (11)	H42A—C42—H42B	108.0
O16—Mo6—O12	94.16 (13)	C44—C43—C42	114.0 (6)
O16—Mo6—O13	152.95 (13)	C44—C43—H43B	108.8
O16—Mo6—O20	88.86 (14)	C42—C43—H43B	108.8
O20—Mo6—O3	75.72 (11)	C44—C43—H43A	108.8
O20—Mo6—O13	79.93 (13)	C42—C43—H43A	108.8
Mo1—O3—Mo2	89.07 (10)	H43B—C43—H43A	107.7
Mo3—O3—Mo1	178.16 (15)	C43—C44—H44B	109.5
Mo3—O3—Mo2	89.09 (10)	C43—C44—H44C	109.5
Mo3—O3—Mo5	90.19 (10)	H44B—C44—H44C	109.5
Mo3—O3—Mo6	90.07 (9)	C43—C44—H44A	109.5
Mo4—O3—Mo1	90.55 (10)	H44B—C44—H44A	109.5
Mo4—O3—Mo2	179.33 (15)	H44C—C44—H44A	109.5
Mo4—O3—Mo3	91.29 (10)	N3—C45—C46	115.6 (4)
Mo4—O3—Mo5	90.48 (10)	N3—C45—H45B	108.4
Mo4—O3—Mo6	91.32 (11)	C46—C45—H45B	108.4
Mo5—O3—Mo1	89.78 (9)	N3—C45—H45A	108.4
Mo5—O3—Mo2	88.97 (10)	C46—C45—H45A	108.4
Mo5—O3—Mo6	178.17 (15)	H45B—C45—H45A	107.4
Mo6—O3—Mo1	89.91 (10)	C49—C46—C45	108.6 (8)
Mo6—O3—Mo2	89.23 (10)	C45—C46—C47	111.4 (7)
Mo3—O9—Mo4	114.15 (14)	C49—C46—H46D	110.0
Mo4—O10—Mo5	114.69 (14)	C45—C46—H46D	110.0
Mo1—O11—Mo4	114.13 (15)	C49—C46—H46C	110.0
Mo6—O12—Mo4	113.99 (14)	C45—C46—H46C	110.0
Mo3—O13—Mo6	116.13 (14)	H46D—C46—H46C	108.4
Mo5—O14—Mo3	116.64 (14)	C45—C46—H46A	109.4
Mo1—O15—Mo5	116.52 (14)	C47—C46—H46A	109.4
Mo6—O16—Mo1	116.47 (14)	C45—C46—H46B	109.4
Mo2—O17—Mo3	117.11 (16)	C47—C46—H46B	109.4
Mo5—O18—Mo2	117.47 (16)	H46A—C46—H46B	108.0
Mo1—O19—Mo2	118.34 (16)	C48—C47—C46	119.8 (11)
Mo2—O20—Mo6	118.01 (15)	C46—C47—H47A	107.4
C1—N1—Mo4	160.7 (5)	C46—C47—H47B	107.4
C1A—N1—Mo4	167.6 (5)	C48—C47—H47A	107.4
N1—C1—C2	117.6 (9)	C48—C47—H47B	107.4
N1—C1—C6	123.4 (9)	H47A—C47—H47B	106.9
C6—C1—C2	118.8 (9)	C50—C49—C46	107.7 (13)
C3—C2—C1	120.5 (10)	C46—C49—H49A	110.2
C3—C2—H2	119.7	C46—C49—H49B	110.2
C1—C2—H2	119.7	C50—C49—H49A	110.2
C4—C3—C2	119.8 (10)	C50—C49—H49B	110.2
C4—C3—H3	120.1	H49A—C49—H49B	108.5
C2—C3—H3	120.1	C49—C50—H50A	109.5
C3—C4—C5	120.9 (11)	C49—C50—H50B	109.5
C3—C4—H4	119.6	C49—C50—H50C	109.5
C5—C4—H4	119.6	H50B—C50—H50C	109.5
C4—C5—C6	120.4 (12)	H50B—C50—H50A	109.5
C4—C5—H5	119.8	H50A—C50—H50C	109.5
C6—C5—H5	119.8	C52—C51—N3	114.8 (4)
C5—C6—C1	119.5 (11)	N3—C51—H51A	108.6
C5—C6—C7	118.0 (10)	N3—C51—H51B	108.6
C1—C6—C7	122.5 (10)	C52—C51—H51A	108.6
O1—C7—O2	123.9 (12)	C52—C51—H51B	108.6
O1—C7—C6	121.2 (11)	H51B—C51—H51A	107.5
O2—C7—C6	115.0 (9)	C51—C52—C53	112.9 (5)
C7—O2—C8	115.7 (10)	C51—C52—H52A	109.0
O2—C8—C9	107.3 (11)	C51—C52—H52B	109.0
O2—C8—H8A	110.3	C53—C52—H52A	109.0
O2—C8—H8B	110.3	C53—C52—H52B	109.0
C9—C8—H8A	110.3	H52B—C52—H52A	107.8
C9—C8—H8B	110.3	C52—C53—H53A	110.9
H8B—C8—H8A	108.5	C52—C53—H53B	110.9
C8—C9—H9A	109.5	C52—C53—H53C	108.1
C8—C9—H9C	109.5	C52—C53—H53D	108.1
H9C—C9—H9A	109.5	C54—C53—C52	104.4 (7)
C8—C9—H9B	109.5	C54—C53—H53A	110.9
H9A—C9—H9B	109.5	C54—C53—H53B	110.9
H9C—C9—H9B	109.5	C55—C53—C52	116.7 (7)
N1—C1A—C6A	125.0 (8)	C55—C53—H53A	131.0
N1—C1A—C2A	115.3 (8)	C55—C53—H53C	108.1
C6A—C1A—C2A	119.7 (8)	C55—C53—H53D	108.1
C3A—C2A—C1A	119.3 (8)	H53A—C53—H53B	108.9
C3A—C2A—H2A	120.4	H53C—C53—H53D	107.3
C1A—C2A—H2A	120.4	C53—C54—H54A	109.5
C4A—C3A—C2A	120.8 (9)	C53—C54—H54B	109.5
C4A—C3A—H3A	119.6	C53—C54—H54C	109.5
C2A—C3A—H3A	119.6	H54B—C54—H54A	109.5
C3A—C4A—C5A	120.3 (10)	H54C—C54—H54A	109.5
C3A—C4A—H4A	119.8	H54B—C54—H54C	109.5
C5A—C4A—H4A	119.8	C53—C55—H55A	109.5
C4A—C5A—C6A	121.1 (9)	C53—C55—H55B	109.5
C4A—C5A—H5A	119.4	C53—C55—H55C	109.5
C6A—C5A—H5A	119.4	H55A—C55—H55B	109.5
C5A—C6A—C1A	118.8 (9)	H55A—C55—H55C	109.5
C5A—C6A—C7A	117.0 (9)	H55B—C55—H55C	109.5
C1A—C6A—C7A	124.1 (8)	N3—C56—C57	120.2 (6)
O1A—C7A—O2A	124.3 (11)	N3—C56—C60	110.3 (5)
O1A—C7A—C6A	122.0 (10)	N3—C56—H56A	109.6
O2A—C7A—C6A	113.7 (8)	N3—C56—H56B	109.6
C7A—O2A—C8A	116.9 (9)	N3—C56—H56C	107.3
O2A—C8A—C9A	111.0 (10)	N3—C56—H56D	107.3
H8AA—C8A—H8AB	108.0	C57—C56—H56C	107.3
O2A—C8A—H8AA	109.4	C57—C56—H56D	107.3
O2A—C8A—H8AB	109.4	C60—C56—H56A	109.6
C9A—C8A—H8AA	109.4	C60—C56—H56B	109.6
C9A—C8A—H8AB	109.4	C60—C56—H56D	140.1
C21—N2—C25	111.4 (3)	H56A—C56—H56B	108.1
C21—N2—C29	106.6 (3)	H56D—C56—H56C	106.9
C21—N2—C33	112.0 (3)	C58—C57—C56	114.3 (11)
C25—N2—C29	110.8 (3)	C56—C57—H57A	108.7
C33—N2—C25	105.1 (3)	C56—C57—H57B	108.7
C33—N2—C29	111.0 (3)	C58—C57—H57A	108.7
N2—C21—C22	116.4 (3)	C58—C57—H57B	108.7
N2—C21—H21B	108.2	H57B—C57—H57A	107.6
N2—C21—H21A	108.2	C59—C58—C57	110.0 (17)
C22—C21—H21B	108.2	C59—C58—H58A	109.7
C22—C21—H21A	108.2	C59—C58—H58B	109.7
H21B—C21—H21A	107.3	C57—C58—H58A	109.7
C23—C22—C21	110.4 (4)	C57—C58—H58B	109.7
C21—C22—H22A	109.6	H58A—C58—H58B	108.2
C21—C22—H22B	109.6	C58—C59—H59A	109.5
C23—C22—H22A	109.6	C58—C59—H59B	109.5
C23—C22—H22B	109.6	C58—C59—H59C	109.5
H22B—C22—H22A	108.1	H59B—C59—H59A	109.5
C24—C23—C22	114.4 (4)	H59C—C59—H59A	109.5
C22—C23—H23A	108.7	H59B—C59—H59C	109.5
C22—C23—H23B	108.7	C56—C60—H60A	110.5
C24—C23—H23A	108.7	C56—C60—H60B	110.5
C24—C23—H23B	108.7	C61—C60—C56	106.4 (7)
H23A—C23—H23B	107.6	C61—C60—H60A	110.5
C23—C24—H24A	109.5	C61—C60—H60B	110.5
C23—C24—H24B	109.5	H60A—C60—H60B	108.6
C23—C24—H24C	109.5	C62—C61—C60	113.7 (11)
H24A—C24—H24B	109.5	C60—C61—H61A	108.8
H24A—C24—H24C	109.5	C60—C61—H61B	108.8
H24B—C24—H24C	109.5	C62—C61—H61A	108.8
C26—C25—N2	116.7 (3)	C62—C61—H61B	108.8
C26—C25—H25A	108.1	H61A—C61—H61B	107.7
N2—C25—H25A	108.1	O103—C102—C101	106.6 (15)
N2—C25—H25B	108.1	O103—C102—H10G	110.4
C26—C25—H25B	108.1	C101—C102—H10G	110.4
H25A—C25—H25B	107.3	O103—C102—H10F	110.4
C25—C26—C27	108.9 (4)	C101—C102—H10F	110.4
C25—C26—H26A	109.9	H10G—C102—H10F	108.6
C25—C26—H26B	109.9	C102—O103—C104	109.1 (16)
C27—C26—H26A	109.9	O103—C104—C105	102.8 (15)
C27—C26—H26B	109.9	O103—C104—H10D	111.2
H26A—C26—H26B	108.3	C105—C104—H10D	111.2
C28—C27—C26	112.5 (4)	O103—C104—H10E	111.2
C26—C27—H27A	109.1	C105—C104—H10E	111.2
C26—C27—H27B	109.1	H10D—C104—H10E	109.1
			
O4—Mo1—O19—Mo2	−179.71 (18)	O20—Mo2—O3—Mo1	93.44 (12)
O15—Mo1—O19—Mo2	−74.02 (19)	O17—Mo2—O3—Mo1	−173.77 (13)
O11—Mo1—O19—Mo2	22.5 (4)	O19—Mo2—O3—Mo1	2.81 (11)
O16—Mo1—O19—Mo2	80.02 (18)	O18—Mo2—O3—Mo1	−84.60 (11)
O3—Mo1—O19—Mo2	3.94 (16)	O6—Mo6—O16—Mo1	−179.33 (17)
O8—Mo2—O19—Mo1	175.2 (2)	O12—Mo6—O16—Mo1	−74.46 (18)
O20—Mo2—O19—Mo1	−80.93 (19)	O20—Mo6—O16—Mo1	76.68 (17)
O17—Mo2—O19—Mo1	3.2 (4)	O13—Mo6—O16—Mo1	11.7 (4)
O18—Mo2—O19—Mo1	72.75 (19)	O3—Mo6—O16—Mo1	1.01 (15)
O3—Mo2—O19—Mo1	−3.93 (16)	O4—Mo1—O16—Mo6	179.14 (19)
O4—Mo1—O11—Mo4	175.01 (16)	O15—Mo1—O16—Mo6	−0.9 (4)
O15—Mo1—O11—Mo4	69.13 (16)	O11—Mo1—O16—Mo6	76.23 (18)
O19—Mo1—O11—Mo4	−27.3 (4)	O19—Mo1—O16—Mo6	−79.04 (18)
O16—Mo1—O11—Mo4	−85.01 (15)	O3—Mo1—O16—Mo6	−1.03 (15)
O3—Mo1—O11—Mo4	−8.68 (13)	O4—Mo1—O15—Mo5	176.53 (18)
N1—Mo4—O11—Mo1	−175.19 (17)	O11—Mo1—O15—Mo5	−78.21 (18)
O10—Mo4—O11—Mo1	−69.95 (16)	O19—Mo1—O15—Mo5	72.61 (19)
O9—Mo4—O11—Mo1	19.6 (4)	O16—Mo1—O15—Mo5	−3.4 (4)
O12—Mo4—O11—Mo1	87.10 (16)	O3—Mo1—O15—Mo5	−3.30 (16)
O3—Mo4—O11—Mo1	9.16 (14)	O7—Mo5—O15—Mo1	−178.03 (19)
O5—Mo3—O9—Mo4	−177.97 (16)	O14—Mo5—O15—Mo1	6.7 (4)
O13—Mo3—O9—Mo4	74.70 (16)	O18—Mo5—O15—Mo1	−75.26 (19)
O17—Mo3—O9—Mo4	−22.2 (4)	O10—Mo5—O15—Mo1	80.01 (18)
O14—Mo3—O9—Mo4	−78.99 (15)	O3—Mo5—O15—Mo1	3.35 (16)
O3—Mo3—O9—Mo4	−3.14 (13)	N1—Mo4—O10—Mo5	−174.82 (16)
N1—Mo4—O9—Mo3	−172.43 (17)	O11—Mo4—O10—Mo5	82.24 (16)
O10—Mo4—O9—Mo3	83.00 (16)	O9—Mo4—O10—Mo5	−73.81 (15)
O11—Mo4—O9—Mo3	−7.2 (4)	O12—Mo4—O10—Mo5	−0.9 (4)
O12—Mo4—O9—Mo3	−74.91 (15)	O3—Mo4—O10—Mo5	4.01 (13)
O3—Mo4—O9—Mo3	3.32 (14)	O7—Mo5—O10—Mo4	179.02 (16)
O6—Mo6—O12—Mo4	177.05 (15)	O14—Mo5—O10—Mo4	74.07 (16)
O16—Mo6—O12—Mo4	70.64 (16)	O18—Mo5—O10—Mo4	−21.6 (3)
O20—Mo6—O12—Mo4	−24.5 (3)	O15—Mo5—O10—Mo4	−80.33 (16)
O13—Mo6—O12—Mo4	−82.25 (15)	O3—Mo5—O10—Mo4	−3.83 (13)
O3—Mo6—O12—Mo4	−6.83 (13)	O8—Mo2—O20—Mo6	174.89 (18)
N1—Mo4—O12—Mo6	−173.72 (17)	O17—Mo2—O20—Mo6	−81.03 (18)
O10—Mo4—O12—Mo6	12.2 (4)	O19—Mo2—O20—Mo6	71.35 (18)
O11—Mo4—O12—Mo6	−72.00 (16)	O18—Mo2—O20—Mo6	−0.7 (4)
O9—Mo4—O12—Mo6	85.89 (16)	O3—Mo2—O20—Mo6	−4.77 (15)
O3—Mo4—O12—Mo6	7.28 (13)	O6—Mo6—O20—Mo2	−179.06 (17)
O7—Mo5—O14—Mo3	−179.84 (17)	O16—Mo6—O20—Mo2	−73.96 (18)
O18—Mo5—O14—Mo3	75.05 (17)	O12—Mo6—O20—Mo2	22.6 (4)
O10—Mo5—O14—Mo3	−76.01 (17)	O13—Mo6—O20—Mo2	81.31 (17)
O15—Mo5—O14—Mo3	−4.7 (4)	O3—Mo6—O20—Mo2	4.81 (15)
O3—Mo5—O14—Mo3	−1.32 (14)	O7—Mo5—O18—Mo2	−175.81 (17)
O5—Mo3—O14—Mo5	−177.62 (18)	O14—Mo5—O18—Mo2	−70.14 (18)
O13—Mo3—O14—Mo5	−6.4 (4)	O10—Mo5—O18—Mo2	24.8 (4)
O9—Mo3—O14—Mo5	79.21 (17)	O15—Mo5—O18—Mo2	83.29 (17)
O17—Mo3—O14—Mo5	−76.96 (17)	O3—Mo5—O18—Mo2	7.26 (15)
O3—Mo3—O14—Mo5	1.35 (15)	O8—Mo2—O18—Mo5	173.04 (18)
O5—Mo3—O13—Mo6	−178.02 (18)	O20—Mo2—O18—Mo5	−11.4 (4)
O9—Mo3—O13—Mo6	−72.28 (18)	O17—Mo2—O18—Mo5	69.86 (18)
O17—Mo3—O13—Mo6	79.47 (18)	O19—Mo2—O18—Mo5	−84.18 (18)
O14—Mo3—O13—Mo6	11.0 (4)	O3—Mo2—O18—Mo5	−7.27 (15)
O3—Mo3—O13—Mo6	3.35 (16)	C21—N2—C29—C30	−167.3 (4)
O6—Mo6—O13—Mo3	176.61 (19)	C33—N2—C29—C30	−45.0 (5)
O16—Mo6—O13—Mo3	−14.3 (4)	C25—N2—C29—C30	71.3 (5)
O12—Mo6—O13—Mo3	74.21 (18)	C21—N2—C25—C26	−51.8 (5)
O20—Mo6—O13—Mo3	−81.21 (19)	C33—N2—C25—C26	−173.3 (4)
O3—Mo6—O13—Mo3	−3.39 (16)	C29—N2—C25—C26	66.7 (5)
O8—Mo2—O17—Mo3	172.49 (19)	C21—N2—C33—C34	59.5 (5)
O20—Mo2—O17—Mo3	68.09 (18)	C25—N2—C33—C34	−179.3 (4)
O19—Mo2—O17—Mo3	−15.5 (4)	C29—N2—C33—C34	−59.6 (5)
O18—Mo2—O17—Mo3	−84.62 (18)	N2—C25—C26—C27	175.1 (4)
O3—Mo2—O17—Mo3	−8.38 (15)	N2—C29—C30—C31	−176.6 (4)
O5—Mo3—O17—Mo2	−176.45 (18)	N2—C33—C34—C35	170.9 (4)
O13—Mo3—O17—Mo2	−70.27 (18)	C29—C30—C31—C32	−168.1 (4)
O9—Mo3—O17—Mo2	27.6 (4)	C25—C26—C27—C28	−180.0 (4)
O14—Mo3—O17—Mo2	84.99 (18)	C56—N3—C41—C42	64.8 (6)
O3—Mo3—O17—Mo2	8.48 (15)	C45—N3—C41—C42	−174.4 (5)
O10—Mo4—O3—Mo3	−93.22 (11)	C51—N3—C41—C42	−53.6 (6)
O11—Mo4—O3—Mo3	173.23 (12)	C56—N3—C45—C46	−59.2 (7)
O9—Mo4—O3—Mo3	−2.43 (10)	C41—N3—C45—C46	−179.8 (6)
O12—Mo4—O3—Mo3	84.82 (11)	C51—N3—C45—C46	58.5 (7)
O10—Mo4—O3—Mo5	−3.01 (10)	C44—C43—C42—C41	72.5 (7)
O11—Mo4—O3—Mo5	−96.56 (11)	N3—C41—C42—C43	−167.8 (5)
O9—Mo4—O3—Mo5	87.77 (11)	C33—C34—C35—C37	168.2 (8)
O12—Mo4—O3—Mo5	175.02 (11)	C33—C34—C35—C36	−85.9 (7)
O10—Mo4—O3—Mo6	176.69 (12)	C33—N2—C21—C22	60.4 (5)
O11—Mo4—O3—Mo6	83.14 (11)	C25—N2—C21—C22	−57.1 (5)
O9—Mo4—O3—Mo6	−92.53 (11)	C29—N2—C21—C22	−178.1 (4)
O12—Mo4—O3—Mo6	−5.27 (10)	N2—C21—C22—C23	177.9 (4)
O10—Mo4—O3—Mo1	86.77 (11)	C56—N3—C51—C52	179.5 (5)
O11—Mo4—O3—Mo1	−6.78 (10)	C41—N3—C51—C52	−59.5 (6)
O9—Mo4—O3—Mo1	177.55 (12)	C45—N3—C51—C52	59.0 (6)
O12—Mo4—O3—Mo1	−95.19 (11)	C21—C22—C23—C24	179.1 (5)
O13—Mo3—O3—Mo4	−93.96 (14)	N3—C51—C52—C53	178.3 (5)
O9—Mo3—O3—Mo4	2.59 (11)	C55—C53—C52—C51	−165.7 (8)
O17—Mo3—O3—Mo4	173.35 (13)	C54—C53—C52—C51	−93.1 (8)
O14—Mo3—O3—Mo4	89.54 (12)	C41—N3—C56—C57	37.4 (9)
O13—Mo3—O3—Mo5	175.55 (14)	C45—N3—C56—C57	−80.6 (9)
O9—Mo3—O3—Mo5	−87.89 (12)	C51—N3—C56—C57	159.1 (8)
O17—Mo3—O3—Mo5	82.87 (12)	C41—N3—C56—C60	80.9 (7)
O14—Mo3—O3—Mo5	−0.95 (10)	C45—N3—C56—C60	−37.2 (7)
O13—Mo3—O3—Mo6	−2.64 (12)	C51—N3—C56—C60	−157.4 (6)
O9—Mo3—O3—Mo6	93.91 (12)	N3—C56—C60—C61	−176.1 (7)
O17—Mo3—O3—Mo6	−95.32 (12)	C57—C56—C60—C61	−62.9 (9)
O14—Mo3—O3—Mo6	−179.14 (13)	N3—C45—C46—C49	156.9 (12)
O13—Mo3—O3—Mo2	86.59 (13)	N3—C45—C46—C47	178.4 (8)
O9—Mo3—O3—Mo2	−176.86 (12)	C48—C47—C46—C49	−102 (3)
O17—Mo3—O3—Mo2	−6.09 (11)	C48—C47—C46—C45	172.1 (10)
O14—Mo3—O3—Mo2	−89.91 (12)	C56—C60—C61—C62	66.5 (12)
O14—Mo5—O3—Mo4	−90.25 (12)	O10—Mo4—N1—C1A	−160 (2)
O18—Mo5—O3—Mo4	174.26 (13)	O11—Mo4—N1—C1A	−65 (2)
O10—Mo5—O3—Mo4	3.00 (10)	O9—Mo4—N1—C1A	109 (2)
O15—Mo5—O3—Mo4	88.19 (12)	O12—Mo4—N1—C1A	23 (2)
O14—Mo5—O3—Mo3	1.04 (11)	O10—Mo4—N1—C1	117 (2)
O18—Mo5—O3—Mo3	−94.45 (12)	O11—Mo4—N1—C1	−148 (2)
O10—Mo5—O3—Mo3	94.29 (12)	O9—Mo4—N1—C1	26 (2)
O15—Mo5—O3—Mo3	179.48 (14)	O12—Mo4—N1—C1	−60 (2)
O14—Mo5—O3—Mo1	179.20 (13)	C1—N1—C1A—C6A	−149 (3)
O18—Mo5—O3—Mo1	83.72 (12)	Mo4—N1—C1A—C6A	95 (2)
O10—Mo5—O3—Mo1	−87.55 (11)	C1—N1—C1A—C2A	33 (2)
O15—Mo5—O3—Mo1	−2.35 (11)	Mo4—N1—C1A—C2A	−84 (2)
O14—Mo5—O3—Mo2	90.13 (12)	N1—C1A—C2A—C3A	177.4 (8)
O18—Mo5—O3—Mo2	−5.36 (11)	C6A—C1A—C2A—C3A	−1.0 (15)
O10—Mo5—O3—Mo2	−176.62 (12)	C1A—C2A—C3A—C4A	−0.3 (14)
O15—Mo5—O3—Mo2	−91.43 (12)	C2A—C3A—C4A—C5A	1.4 (16)
O16—Mo6—O3—Mo4	−91.34 (13)	C3A—C4A—C5A—C6A	−1.2 (15)
O12—Mo6—O3—Mo4	5.72 (10)	C4A—C5A—C6A—C1A	−0.1 (14)
O20—Mo6—O3—Mo4	176.98 (13)	C4A—C5A—C6A—C7A	−179.9 (10)
O13—Mo6—O3—Mo4	93.69 (12)	N1—C1A—C6A—C5A	−177.1 (9)
O16—Mo6—O3—Mo3	177.37 (14)	C2A—C1A—C6A—C5A	1.2 (14)
O12—Mo6—O3—Mo3	−85.57 (11)	N1—C1A—C6A—C7A	2.7 (17)
O20—Mo6—O3—Mo3	85.68 (12)	C2A—C1A—C6A—C7A	−179.0 (10)
O13—Mo6—O3—Mo3	2.40 (11)	C5A—C6A—C7A—O1A	10.0 (16)
O16—Mo6—O3—Mo1	−0.80 (12)	C1A—C6A—C7A—O1A	−169.8 (11)
O12—Mo6—O3—Mo1	96.27 (12)	C5A—C6A—C7A—O2A	−170.7 (9)
O20—Mo6—O3—Mo1	−92.48 (12)	C1A—C6A—C7A—O2A	9.5 (16)
O13—Mo6—O3—Mo1	−175.76 (14)	O1A—C7A—O2A—C8A	1.3 (18)
O16—Mo6—O3—Mo2	88.28 (12)	C6A—C7A—O2A—C8A	−178.1 (9)
O12—Mo6—O3—Mo2	−174.66 (12)	C7A—O2A—C8A—C9A	76.1 (15)
O20—Mo6—O3—Mo2	−3.41 (10)	C1A—N1—C1—C6	18.7 (17)
O13—Mo6—O3—Mo2	−86.69 (12)	Mo4—N1—C1—C6	163.2 (11)
O15—Mo1—O3—Mo4	−87.90 (13)	C1A—N1—C1—C2	−157 (4)
O11—Mo1—O3—Mo4	6.99 (11)	Mo4—N1—C1—C2	−13 (3)
O19—Mo1—O3—Mo4	177.71 (14)	N1—C1—C2—C3	175.3 (10)
O16—Mo1—O3—Mo4	92.04 (12)	C6—C1—C2—C3	−0.8 (19)
O15—Mo1—O3—Mo5	2.58 (12)	C1—C2—C3—C4	−1.1 (17)
O11—Mo1—O3—Mo5	97.46 (12)	C2—C3—C4—C5	1.1 (18)
O19—Mo1—O3—Mo5	−91.81 (13)	C3—C4—C5—C6	1.0 (19)
O16—Mo1—O3—Mo5	−177.48 (13)	C4—C5—C6—C1	−2.9 (19)
O15—Mo1—O3—Mo6	−179.22 (14)	C4—C5—C6—C7	178.5 (11)
O11—Mo1—O3—Mo6	−84.33 (12)	N1—C1—C6—C5	−173.1 (12)
O19—Mo1—O3—Mo6	86.39 (13)	C2—C1—C6—C5	2.8 (19)
O16—Mo1—O3—Mo6	0.72 (11)	N1—C1—C6—C7	5 (2)
O15—Mo1—O3—Mo2	91.55 (13)	C2—C1—C6—C7	−178.7 (11)
O11—Mo1—O3—Mo2	−173.57 (12)	C5—C6—C7—O1	24.4 (17)
O19—Mo1—O3—Mo2	−2.84 (12)	C1—C6—C7—O1	−154.1 (12)
O16—Mo1—O3—Mo2	−88.51 (12)	C5—C6—C7—O2	−155.5 (12)
O20—Mo2—O3—Mo3	−86.56 (12)	C1—C6—C7—O2	25.9 (17)
O17—Mo2—O3—Mo3	6.23 (12)	O1—C7—O2—C8	−0.5 (18)
O19—Mo2—O3—Mo3	−177.18 (13)	C6—C7—O2—C8	179.5 (10)
O18—Mo2—O3—Mo3	95.40 (12)	C7—O2—C8—C9	152.8 (12)
O20—Mo2—O3—Mo5	−176.77 (12)	C102—O103—C104—C105	−168 (2)
O17—Mo2—O3—Mo5	−83.98 (12)	C104—O103—C102—C101	−180 (2)
O19—Mo2—O3—Mo5	92.61 (12)	N3—C56—C57—C58	−165.0 (12)
O18—Mo2—O3—Mo5	5.19 (10)	C60—C56—C57—C58	108.8 (16)
O20—Mo2—O3—Mo6	3.52 (11)	C56—C57—C58—C59	−169.8 (13)
O17—Mo2—O3—Mo6	96.31 (12)	C45—C46—C49—C50	−172.0 (13)
O19—Mo2—O3—Mo6	−87.10 (12)	C47—C46—C49—C50	87 (3)
O18—Mo2—O3—Mo6	−174.52 (12)		

### Hydrogen-bond geometry (Å, º)

**Table:**

*D*—H···*A*	*D*—H	H···*A*	*D*···*A*	*D*—H···*A*
C3*A*—H3*A*···O19^i^	0.93	2.38	3.287 (9)	164
C5—H5···O7^ii^	0.93	2.58	3.449 (14)	155
C21—H21*B*···O15^i^	0.97	2.43	3.354 (5)	159
C25—H25*A*···O6^iii^	0.97	2.58	3.544 (5)	173
C41—H41*B*···O14	0.97	2.56	3.480 (6)	158
C44—H44*B*···O8^i^	0.96	2.57	3.495 (8)	162
C45—H45*A*···O1*A*^ii^	0.97	2.45	3.345 (8)	153
C45—H45*A*···O1^ii^	0.97	2.22	3.103 (9)	151
C32—H32*B*···O13	0.96	2.54	3.387 (6)	147
C3—H3···O19^i^	0.93	2.47	2.986 (10)	115
C37—H37*A*···O4^iii^	0.96	2.60	3.173 (14)	118
C5*A*—H5*A*···O7^ii^	0.93	2.49	3.133 (9)	126
C9—H9*C*···O11	0.96	2.54	3.25 (2)	130
C9*A*—H9*AC*···O11	0.96	2.48	3.262 (10)	138

Symmetry codes: (i) *x*−1/2, −*y*+3/2, *z*−1/2; (ii) −*x*, −*y*+1, −*z*+1; (iii) −*x*, −*y*+2, −*z*+1.
